# Adaptive optics in laser processing

**DOI:** 10.1038/s41377-019-0215-1

**Published:** 2019-11-29

**Authors:** Patrick S. Salter, Martin J. Booth

**Affiliations:** Department of Engineering Science, University of Oxford, Parks Road, Oxford, OX1 3PJ UK

**Keywords:** Laser material processing, Adaptive optics, Adaptive optics, Laser material processing

## Abstract

Adaptive optics are becoming a valuable tool for laser processing, providing enhanced functionality and flexibility for a range of systems. Using a single adaptive element, it is possible to correct for aberrations introduced when focusing inside the workpiece, tailor the focal intensity distribution for the particular fabrication task and/or provide parallelisation to reduce processing times. This is particularly promising for applications using ultrafast lasers for three-dimensional fabrication. We review recent developments in adaptive laser processing, including methods and applications, before discussing prospects for the future.

## Optics: Adaptive optics key to expanding laser fabrication applications

The integration of adaptive optics with laser processing is enhancing the capabilities of laser systems, enabling precision fabrication inside materials, the tailoring of focal intensity for structured and wide-area fabrication, and fine control of the fabrication process. Patrick Salter and Martin Booth from the University of Oxford in the United Kingdom have undertaken a review of how recent developments in adaptive optics are providing advanced control of the laser fabrication process. They show how simple adaptive elements, such as liquid crystal spatial light modulators and digital micromirror devices, which allow for the phase, amplitude, and polarisation of the fabrication beam to be modulated, are providing many possibilities for advanced control of the laser fabrication process, and, as the technology develops further, the range of applications of adaptive optical laser fabrication is likely to expand.

## Introduction

Over the past two decades, direct laser writing (DLW) with ultrafast lasers has developed into a mature, diverse and industrially relevant field^1–5^. The ultrashort nature of the laser pulses means that energy can be delivered to the focus in a period shorter than the characteristic timescale for thermal diffusion, leading to highly accurate material modification^1^. Thus, by focusing ultrashort pulses onto the surface of the workpiece, precise cuts and holes can be manufactured with a minimal heat-affected zone. However, the DLW technique has further functionality, as when pulses are focused inside a transparent material, the electric field at the focus is sufficiently high for multi-photon absorption or other non-linear effects allowing internal structuring^6^. The highly nonlinear nature of the interaction causes the material modification to be confined entirely to the focus, opening the door to three-dimensional fabrication. A wide range of materials are accessible, including many different plastics, glasses and crystals. Advances have been made in areas such as telecommunications, lab-on-chip, quantum technology and astrophotonics, to name a few. Industrial adoption of ultrafast lasers is equally diverse^7,8^ in fields such as ophthalmology, glass cutting, and the manufacture of fibre-based sensors.

As the field progresses, there is increasing interest and uptake in the use of adaptive optics (AO) techniques to enhance ultrafast DLW. An adaptive optical element enables control over the fabrication laser beam and allows it to be dynamically updated during processing. Adaptive elements can modulate the phase, amplitude and/or polarisation of the fabrication beam, providing many possibilities for advanced control of the laser fabrication process. In this review, we briefly outline the application areas of AO for laser processing before considering the methods of AO, including the range of adaptive elements that are available for use in DLW. This is followed by some demonstrations of the benefits of AO in DLW applications. We conclude with a discussion of future prospects in this area.

## Applications of AO

The integration of AO with laser fabrication can bring many benefits, either through control over the spatial and temporal intensity distribution at the laser focus or through an adaptive parallelisation of the process using arrays of foci to significantly reduce the processing times.

**Fig. 1:**
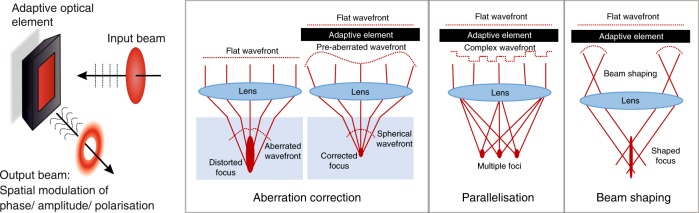
Adaptive optical elements allow spatial shaping of a laser beam in phase, amplitude and/or polarisation. This offers great flexibility for aberration correction, focal shaping and parallelisation in laser processing.

Addressing first the concept of focal intensity control, adaptive aberration correction provides an instructive example. Optical aberrations disrupt the focusing of light such that the wavefronts no longer converge in phase to a single point. Thus, aberrations introduce focal distortion, changing the form of the focal intensity distribution from its ideal diffraction-limited nature, as shown in Fig. 1. This can be particularly problematic in laser processing, where the dimensions of the material modification are closely matched to the three-dimensional shape of the laser focus. Aberrations are generated by a non-uniform refractive index distribution, which occurs mainly in laser processing at the interface of the workpiece. If the phase aberration introduced by focusing inside the sample is known, the opposite phase can be imposed on the laser beam by an adaptive optical element positioned prior to the objective lens, thus cancelling the aberration on propagation to the focus, as illustrated in Fig. 1. Thus, through aberration correction, it is possible to maintain a system at diffraction-limited performance and hence give optimum operation anywhere throughout a three-dimensional sample. The adaptive nature of the optical element is critical since a different phase correction is required at each fabrication location in 3D.

In the absence of optical aberrations, adaptive optical elements are still a useful resource, providing the capability of complex shaping of the laser focus. Since the shape of the laser-induced structural modifications is related to that of the laser focus, there exist many opportunities for shaping the laser focus to tune the form of the fabricated features for particular device applications. Adaptive optical elements are useful tools for controlling the shape of laser foci, allowing spatially complex phase and amplitude distributions of light to be generated at the pupil plane of the focusing objective lens, giving rise to much flexibility in the form of the intensity at the focus, as seen in Fig. 1. The adaptive nature of the element allows the beam shaping to be tuned during fabrication to give different-shaped features at different parts of a device.

Adaptive optical elements additionally help to address one of the key challenges for DLW: the point-based nature of the process can lead to long processing times, especially for high-resolution three-dimensional patterning of large volumes. Modern femtosecond lasers often have significantly more pulse energy than is needed for fabrication at a single focus, offering many opportunities for parallelisation. Adaptive optical elements may be used to split a beam and generate multiple foci simultaneously through a single lens for parallel processing^9–14^, as shown in Fig. 1. Processing times can be reduced dramatically through the use of multiple foci, while the reconfigurability of the element means that the spot pattern can be updated during processing to form large complex structures.

## Adaptive elements

There exists a range of reconfigurable optical elements that can change the properties of a beam across its profile. This can involve modification of the phase, amplitude or polarisation of the beam. These devices are essential components of any AO system. We outline in this section the options commonly available and important considerations for their use in DLW.

### Liquid crystal spatial light modulators (SLMs)

Liquid crystal (LC)-based SLMs are the most commonly used adaptive elements, in part due to their ease of use, flexibility in operation and high resolution. While some early models were transmission devices, the vast majority now operate in reflection mode with a liquid crystal layer on a silicon (LCOS) back plane. Predominantly, these devices use nematic liquid crystals and are designed to provide phase-only modulation, but dependent on the orientation of the polarisation state of the incident laser, these SLMs can be used to control the amplitude, phase and/or polarisation of the output beam.

SLM devices operating in the nematic phase allow continuous modulation of the output state of the laser beam. They are generally restricted to switching rates in the tens of hertz and become slower for longer wavelengths due to a necessary increase in the device thickness. However, it has been shown that, using overdrive techniques, it is possible to approach modulation rates of 1 kHz, still in the nematic phase with greyscale modulation^15^. SLMs that employ a ferroelectric liquid crystal phase can operate much faster, at rates above 1 kHz, but are restricted to binary modulation.

A typical configuration for laser processing uses a nematic SLM to give continuous phase-only control over the laser fabrication beam. The phase modulation range is limited to [0, 2π] radians by the thickness of the LC layer, but much greater ranges of phase modulation may be achieved by wrapping the phase from 2π → 0 radians between adjacent pixels. The ability to create discontinuities in the phase between adjacent pixels provides further functionality for beam shaping and parallelisation. As LC SLMs are usually not perfectly flat when manufactured, it is necessary to display an appropriate phase flattening pattern as a background to compensate for any phase errors introduced by the SLM itself^16,17^.

### Digital mirror devices (DMDs)

DMDs are based on a MEMS platform and have been developed extensively for optical projector technology. They consist of a micro-mirror at each pixel that can be rapidly switched between two orientations, offering amplitude modulation by directing light into or out of the laser beam path. High-speed switching is achievable, with rates up to 40 kHz, and the units tend to be low cost and easy to control. Limitations relate to the binary nature of the amplitude modulation, which has lower efficiency than phase modulation. Additionally, use with very short pulses is constrained by the dispersion generated by the device: when all pixels are in the “on” position, the device forms a uniform blazed diffraction grating with a pitch of approximately 5 µm, the angular dispersion of which significantly affects pulses with durations less than approximately 1 ps. It should be additionally noted that current DMDs have average power limitations of approximately 25 W due to the low thermal mass of the micro-mirrors.

### Deformable mirrors (DMs)

DMs consist of a highly reflective surface whose shape can be controlled by the application of forces from an array of actuators. The surface may be a continuous membrane or arranged into segments, and the mirror may be actuated by electrostatic, electromagnetic or piezoelectric means. DMs are insensitive to polarisation and have good reflectance over a wide range of wavelengths. They typically have tens to hundreds of actuators, which is much smaller than the typical number of pixels for an SLM or a DMD. Commercially available mirrors can handle beam powers of a few watts for expanded beams, although it is possible to make devices that can handle much higher beam powers, such as DMs that are used in high power laser systems.

### Optical system configuration

While the details of the optical system for adaptive laser machining will vary for each application, there are some general principles for which we provide an overview here.

In all of these systems, the adaptive element will be placed before the objective lens to modulate the light before focusing. Normally, the incoming laser beam will be expanded to fill the active area of the adaptive element; indeed, in laser machining applications, it is important to ensure that the adaptive element is not placed near a focus, otherwise, the laser could cause damage to the device. The vast majority of adaptive optical elements are reflective in design. In this case, the adaptive element is usually placed such that the incident and reflected beams are close to but slightly off normal incidence (preferably less than 10° with respect to the normal). Normal incidence configurations are also possible but require beamsplitters and possibly polarisation elements, which could lead to extra complexity and losses. With transmissive devices, a simpler inline arrangement can be used.

In all of these configurations, it is important to note that the adaptive element must be imaged onto the plane of interest in the optical system, which will typically be the pupil plane of the focusing objective lens. Without an imaging stage, diffraction occurs due to the phase and/or amplitude distribution imposed on the fabrication beam by the adaptive element, distorting the beam profile as a function of propagation distance. A faithful reproduction of both the amplitude and phase at the adaptive element is required in the pupil plane. For this reason, a 4*f* imaging system should be employed. Note that while a single lens would create a faithful image of the intensity profile at the pupil; the phase would not be reproduced, as there would be an additional quadratic phase variation across the image. Relay lenses of different focal lengths may be employed in a 4*f* configuration to provide the necessary magnification between the adaptive element and the objective pupil. It is important to utilise as much of the active area in the adaptive element as possible while still filling the aperture of the objective. An intensity modulation filter, such as a pin hole or beam block, is sometimes inserted into the Fourier plane of the adaptive element to prevent the zero-order, or unmodulated light from the adaptive element, from propagating through the system to the workpiece. This is especially relevant to parallelisation applications and is discussed further in Section 4.2.

## Implementations of AO in DLW

### Aberration correction

Optical aberrations cause focal distortion leading to loss of resolution and efficiency in laser fabrication. Spatial variation in the aberration leads to non-uniformity across a laser processed device and loss of functionality. The most problematic aberrations in laser processing are related to variation in the refractive index along the optical path to the laser focus, particularly at the surface of the workpiece^18–22^. When focusing inside a sample through a planar interface, refraction at the surface generates a spherical aberration, the strength of which is related to the numerical aperture of the lens, the refractive index difference at the surface and the focusing depth^23^. The spherical aberration leads predominantly to distortion of the focus along the optical axis and can severely limit the potential for accurate 3D laser fabrication. Even in situations where the shape of the focus and laser-modified zone are largely decoupled, such as in the cumulative heating regime in glasses, the effect of aberrations is found to be problematic and can affect the quality of the fabricated devices^24,25^. If the refractive index interface focused through for fabrication is not planar but curved as, for example, in an optical fibre, the aberrations are significantly more complex^26^. Further aberrations commonly encountered in laser processing occur at the edge of samples, for example, when writing transverse waveguides, and relate to portions of the fabrication laser focusing through different facets of the sample, leading to focal splitting^27^. Other causes of focal splitting are related to focusing inside birefringent media, where different polarisation modes couple to different refractive indices^28–30^. In general, aberrations degrade the performance of laser processing and are increasingly problematic at higher numerical apertures.

In general optical systems, the determination of the aberrations can be highly complicated^31^, but for laser processing applications, it can be simplified since often only the depth-dependent spherical aberration is important. By mapping rays of light from the focus back to the pupil plane of the objective lens, an analytic expression may be derived for the phase of the depth-dependent spherical aberration^23^. Thus, an adaptive optical element placed in a plane conjugate to the objective pupil may be used to apply the opposite phase, providing a predictive aberration correction, which is sufficient in many circumstances^32^. If a more precise level of aberration correction is required, a method of focal feedback is necessary to optimise the correction phase, with techniques including optimisation of the focal plasma intensity^33^, measurements of the focal intensity distribution^34^, live phase imaging^35^ or a modal approach to find the phase distribution that minimises the laser power fabrication threshold^36^.

**Fig. 2:**
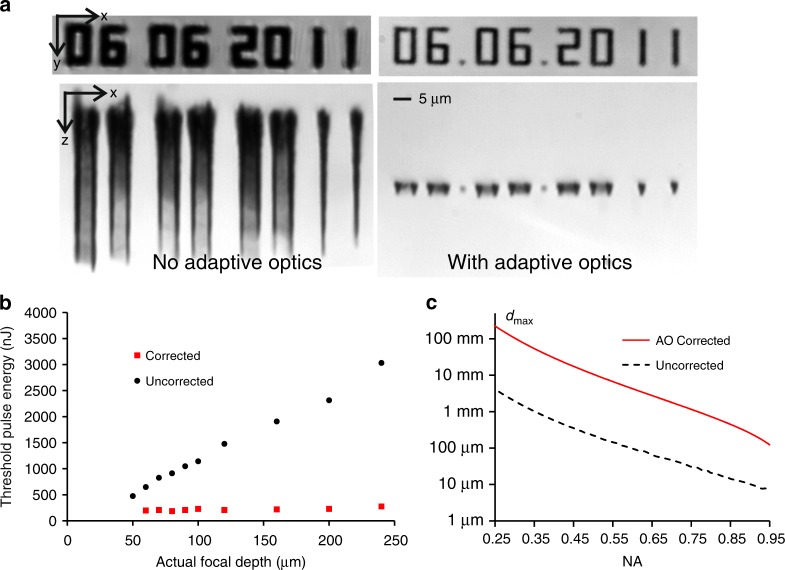
Adaptive Optics aberration correction of depth-dependent spherical aberration. **a** Ultrafast laser fabrication beneath the surface of diamond without and with aberration correction. The laser was incident along the z direction^36^. **b** Variation in the threshold pulse energy for laser fabrication as a function of depth in diamond^36^. **c** Simulated maximum fabrication depth *d*_max_ for diffraction-limited performance as a function of the numerical aperture (NA) with and without adaptive optics aberration correction. **a**, **b** Reproduced from ref. ^36^ with permission from OSA.

Figure 2 demonstrates some practicalities of aberration correction in laser processing. The transmission microscope images in Fig. 2a show ultrashort pulse laser fabrication beneath the surface of diamond, with the fabrication laser incident along the z axis. Without AO, there is severe spherical aberration due to a high NA of 1.4 and a large refractive index mismatch at the surface of the sample. Therefore, the laser-induced modifications from the aberrated focus display characteristic elongation along the optic axis. When AO aberration correction is applied, accurate and controlled fabrication of structures with axial confinement becomes possible^36^. A benefit of aberration compensation is that the threshold pulse energy for fabrication is invariant as a function of depth inside the sample, as demonstrated in Fig. 2b. Indeed, invariance of the fabrication threshold with depth can be used as evidence for a well-functioning adaptive optical aberration correction system. As stated above, the objective lens NA has a strong influence on the depth-dependent spherical aberration. Figure 2c shows the maximum depth that can be accessed for fabrication in glass using air-based objective lenses while maintaining diffraction-limited performance as a function of the numerical aperture. At 0.95 NA, the maximum depth is expected to be below 10 µm. Through characterisation of adaptive optical elements^37^, it is possible to estimate the extent to which the maximum fabrication depth can be extended^38^, yielding an improvement of approximately two orders of magnitude. In many cases, this is approaching or exceeding the working distance limit of the objective lens.

**Fig. 3:**
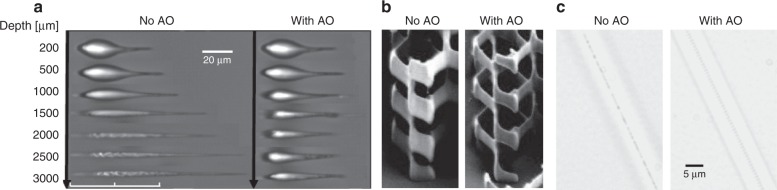
Examples of laser fabrication without adaptive optics (No AO) and using adaptive optics for aberration compensation (With AO). **a** Images showing laser fabrication deep inside a glass workpiece, where the AO leads to more uniform fabrication and reduced axial elongation^35^. **b** Gyroid photonic crystals fabricated inside high-refractive-index chalcogenide materials^39^. **c** Laser writing through a cylindrical interface for fabrication of fibre Bragg gratings with air-based optics loses all resolution without the appropriate use of AO aberration compensation^26^. Images reproduced from **a** ref. ^35^, **b** ref. ^39^ and **c** ref. ^26^ with permission from OSA.

Figure 3 provides some further examples of systems successfully exploiting aberration correction for accurate high-resolution fabrication using a range of different materials and processing conditions. Figure 3a shows photonic waveguides written inside glass using a longitudinal writing geometry, where the waveguide axis is along the direction of substrate motion^35^. At large depths of up to 3 mm with a focusing NA of 0.45, the aberration can be compensated using a phase microscopy feedback system. Figure 3b shows laser-written gyroid photonic crystals fabricated from chalcogenide materials at high NA for maximum resolution^39^. The clear reduction in axial extent of the fabricated features when using AO is symptomatic of compensation for spherical aberration. Figure 3c demonstrates the benefits of aberration compensation when the sample interface is not planar. The images show the fabrication of fibre Bragg gratings inside the core of an optical fibre using air-based focusing optics, where the AO correct for a strong astigmatism generated by the cylindrical geometry of the fibre interface^26^.

**Fig. 4:**
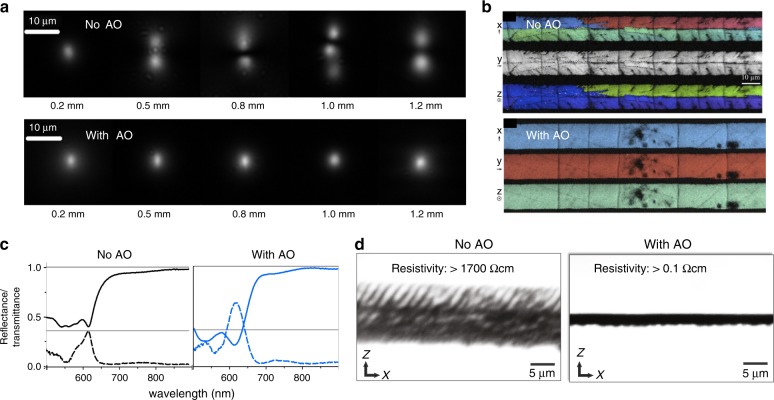
AO aberration correction during laser fabrication can provide significant improvements in the operation of devices. **a** Images of the propagating mode in photonic waveguides written at different depths in glass, where without AO, the waveguides become increasingly multimode with the depth^40^. **b** Selective laser-induced crystallisation in glasses, where AO aberration compensation is needed to form uniform single-crystal waveguides^47^. **c** Photonic crystal fabrication via two-photon polymerisation, with AO leading to enhanced spectral performance^49^. **d** Laser-written graphitic wires beneath the surface of diamond, with the resistivity dropping dramatically with aberration compensation^54^. Images reproduced from **a** ref. ^40^ with permission from OSA, **b** ref. ^47^ with permission from Springer, **c** ref. ^49^ with permission from OSA and **d** ref. ^54^ with permission from AIP.

Aberration correction using AO can be highly beneficial in the laser-based manufacturing of three-dimensional devices. Indeed, this three-dimensional capability has often been cited as an important benefit of DLW in the writing of photonic waveguide circuits, yet this is rarely fully exploited due to complications with depth-dependent aberrations. Recent demonstrations have shown that aberration correction can maintain single-mode waveguide operation over depth ranges greater than 1 mm^24,35,40^, as shown in Fig. 4a, and lead to more controlled coupling in large photonic lattices^25,41,42^. Work has also been extended to showcase the benefits of aberration correction in laser-written waveguides in high-index and/or birefringent crystals such as lithium niobate^43^, KDP^44^ and diamond^45,46^. The depth-dependent aberration can also be highly detrimental for laser-induced crystallisation inside specialty glasses for applications writing non-linear single-crystal-in-glass waveguides. As shown in Fig. 4b, when aberration compensation is applied, it is possible to accurately pattern mono-domain single-crystal structures^47,48^.

Aberrations are especially critical when fabricating at the highest resolution, as is the case in two-photon polymerisation of photonic crystals. As shown in Fig. 4c, AO aberration correction can significantly enhance the spectral characteristics of devices^49^ and has been used in a variety of materials, including lithium niobate^50^ and chalcogenides^39,51–53^. Aberration correction has furthermore been transformative for writing 3D conductive graphitic wires beneath the surface of diamond, as shown in Fig. 4d^54^, where the resistivity of the wires drops by orders of magnitude when using AO, creating possibilities for the fabrication of radiation detectors^55^ and components for quantum processing^56–58^. Aberration correction has also been successfully applied to different fabrication strategies for laser-written fibre Bragg gratings^26,59^, three-dimensional data storage in polymers^32^, tailored edge-cleaving of glass plates^60^, and fabrication inside liquid crystal devices^61,62^.

### Beam shaping

**Fig. 5:**
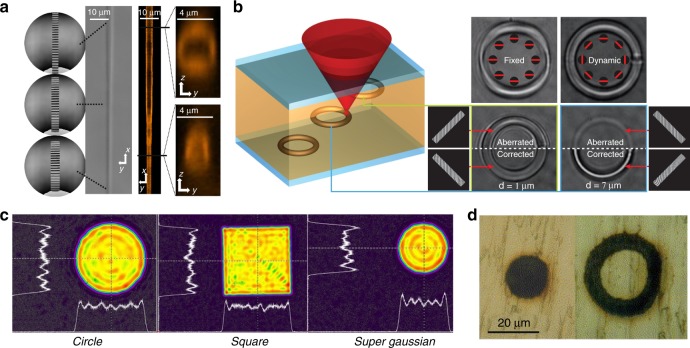
Adaptive Optics for shaping the focal intensity distribution of the fabrication laser. Adaptive slit beam shaping during laser fabrication for **a** photonic waveguides of varying cross-section^65^ and **b** two-photon polymerisation of curved structures^66^. The use of adaptive optical elements for generating flat top beam intensity profiles (**c**)^72^, as used in laser ablation (**d**)^74^. Images reproduced from **a** ref. ^65^ with permission from OSA, **b** ref. ^66^ with permission from OSA, **c** ref. ^72^ with permission from SPIE and **d** ref. ^74^ with permission from Elsevier.

Beam shaping brings flexibility to the morphology of features induced by laser fabrication such that they are not limited in shape to the prolate ellipsoid typical of a diffraction-limited laser focus. A simple, but effective, example of adaptive laser beam shaping is given in Fig. 5a. Laser fabrication foci have an elliptical cross-section, with an extended length along the optical axis. When machining tracks inside a material transverse to the optic axis, the track cross-section mirrors the elliptical shape of the diffraction-limited focus. This asymmetry is problematic when trying to create structures with circular cross-sections, such as for waveguide^63^ and microfluidic^64^ devices, among others. A convenient solution to this problem is to place a slit before the focusing objective lens to spread the focal intensity in a direction perpendicular to the axis of the slit while maintaining the axial resolution. This produces a disc-like focus that can be utilised to form symmetric laser-induced modifications^63,64^. Using an adaptive optical element to generate the slit illumination presents some advantages, as shown in Fig. 5, since during fabrication, the slit may be rotated to maintain appropriate alignment of the focal disk to curved tracks; alternatively, the slit dimensions can be altered to tune the cross-section profile^65–68^. An alternative strategy uses an astigmatic beam to control the laser-writing cross-section^69^ and has also been implemented using adaptive elements for increased flexibility^70,71^.

Another area of great interest for beam shaping using AO is the generation of a “flat top” intensity profile. Typically, when a beam is focused, it displays a Gaussian or Airy disc intensity profile. In certain processes, the associated laser fabrication can display a variation in strength across the modified region in direct relation to the intensity profile, while a modification of uniform cross-section is desired. Thus, there has been much effort in spatial beam shaping to generate a focus with a flat top intensity profile, displaying a uniform intensity over the majority of its cross-section with a rapid transition to low intensity at the edges^72,73^, as shown in Fig. 5c. The flat top beams generated using AO have been successfully used for surface machining in a wide variety of materials^74–77^ (Fig. 5d). The extra flexibility offered by AO is useful in tailoring the beam shaping to a specific experimental system^78^. This beam shaping is invaluable in large area processing requiring high uniformity but also finds application at the nanoscale, such as in the controlled fabrication of silver nanowires^79^.

**Fig. 6:**
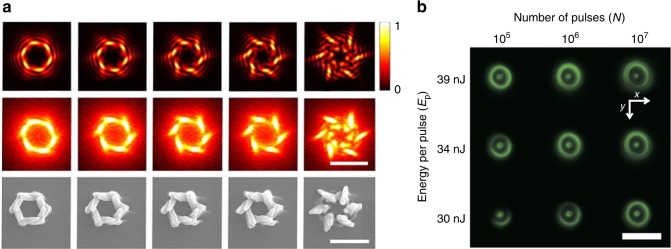
Laser fabrication using adaptive optics to generate the OAM to shape the fabrication focus. **a** Two-photon polymerisation using the OAM to generate a chiral surface^85^. Scale bar: 5 µm. **b** STED-inspired fabrication of nanoscale structures inside a photo-responsive glass^95^. Scale bar: 4 µm. Images reproduced from **a** ref. ^85^ with permission from AIP and **b** ref. ^95^ with permission from OSA.

There is currently great interest in the manipulation of the orbital angular momentum (OAM) of a beam^80^, and this has driven research using the OAM to shape the beam focus during DLW, both in two-photon polymerisation^81^ and in surface^82,83^ and bulk^84^ structuring of solid dielectrics. Adaptive elements are used to generate varying states of OAM for different focal intensity distributions, creating a myriad of complex laser-written modifications (Fig. 6a)^85–90^. Furthermore, since it was shown that super-resolution fabrication could be achieved using a stimulated emission depletion (STED) inspired process for two-photon polymerisation of sub-30 nm structures^91–94^, there has been a recent implementation of AO to generate the depletion focus^95^ (Fig. 6b), accounting for system aberrations to give higher-fidelity fabrication^34,96^.

**Fig. 7:**
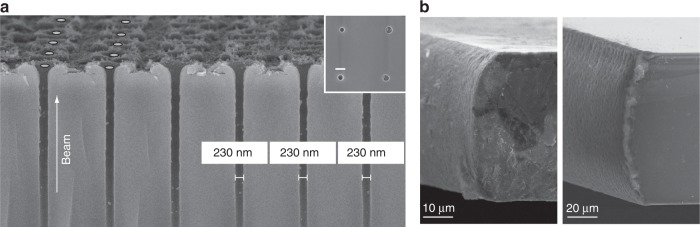
Using adaptive optics to generate foci with extended depth of field. **a** Bessel beam laser processing of nanoscale holes in glass^104^, and **b** Airy beam laser dicing of diamond (left) and silicon (right) with a curved chamfer^113^. Images reproduced from **a** ref. ^104^ and **b** ref. ^113^ with permission from AIP.

A major new avenue for beam shaping with adaptive elements in laser fabrication comes with the uptake of Bessel beams for a variety of laser processing tasks^97–100^. AO are often used to shape light with a conical wavefront to create a Bessel beam in an equivalent way to that for an axicon^101^ but with a greater level of flexibility and control^102^. The light distribution is characterised as a central spot with a diameter below that for an Airy focus of equivalent NA but an extended depth of field that can stretch for several hundreds of micrometres with a uniform axial intensity^103^. These beams have been demonstrated to be efficient tools for generating vias through wafers with extraordinary aspect ratios exceeding 100:1^104^. Figure 7a shows an array of 200 nm holes in a fused silica wafer created through single-pulse machining with a Bessel beam^104^. Successful demonstrations have been given for a range of other materials, including different glasses^105,106^, sapphire^107^, silicon^103^ and diamond^108^. Bessel beams have also been applied to engineer microfluidic channels with sharp sidewalls^109^ and to remove the need for focal translation in the surface processing of samples with complex topographies^110^. There are industrial applications in cutting and dicing tasks and the potential to provide new perspectives on the fundamental physics of light-matter interactions^111^. There is interesting potential for other exotic foci with an extended depth of field, such as the Airy beam^112^. The Airy beam creates a focal intensity distribution that follows a curved caustic, appearing to curve as the light propagates, and has been used to generate curved chamfers on silicon and diamond wafers, as shown in Fig. 7b^113^. Recently, tuneable ring-Airy beams with abrupt autofocusing properties have been demonstrated, with great potential for fabrication of large 3D structures by extending the working distance of available optics^114^.

### Parallelisation

**Fig. 8:**
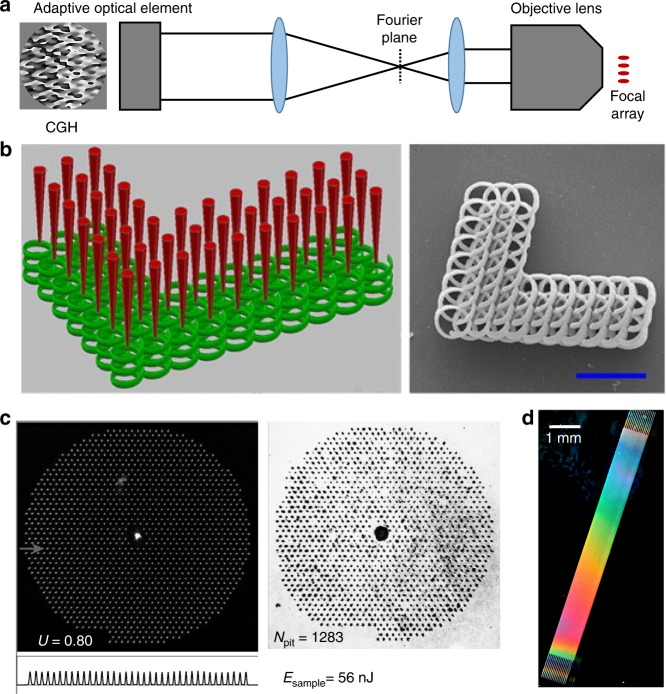
Multiple foci generated by adaptive optics to reduce processing times. **a** Parallelisation of laser processing using an adaptive element to display a computer-generated hologram (CGH) that is imaged onto the pupil plane of the objective lens to create an array of spots at the focus. **b** Simultaneous two-photon polymerisation of complex scaffold structures from 42 focal spots (scale bar: 20 µm)^119^. **c** Massive parallelisation using an SLM to generate 1283 foci for fabrication on the surface of silicon^126^. **d** A large 3D void array in fused silica, containing 64 million voxels with a lattice spacing of 2.5 µm, enabled through parallel fabrication using a 196 spot focal array. Images reproduced from **b** ref. ^119^ with permission from Elsevier and **c** ref. ^126^ with permission from OSA.

Parallelisation can bring significant reductions in laser processing times by ensuring that more of the available laser power is effectively employed. Adaptive elements are powerful tools for parallelisation, providing tuneable and reconfigurable splitting of the fabrication laser beam. A common method for splitting the beam to generate an array of foci for laser fabrication is to display a diffractive pattern (hologram) on an SLM using phase and/or amplitude modulation. Computational techniques such as the Gerchberg-Saxton algorithm^115,116^, ORA^117^, direct binary search or multiplexing Fresnel lenses^118^ are used to generate the hologram. As shown in Fig. 8a, the hologram displayed on the adaptive element, which is usually an SLM, is imaged onto the pupil plane of the objective, creating an array of spots in the focal plane. The spot array can then be used to simultaneously fabricate many features at once, as shown in the examples of Fig. 8, to reduce the processing times by more than an order of magnitude^119^. As the light-matter interaction is highly nonlinear, it is very important to have a high degree of intensity uniformity across the shaped light field^120–122^. In some cases, computational optimisation alone is not sufficient, and further experimental feedback is required to increase uniformity^123–125^. Massive parallelisation can thus be realised, with recent demonstrations creating arrays with more than 1000 individual fabrication foci (Fig. 8c)^126^. The focal array can then form a building block that can be tiled together to make macroscale structures of closely spaced micron scale features, as in the centimetre scale void array shown in Fig. 8d, which was printed using a 196 focal spot array.

The imperfect way in which an SLM reproduces the phase patterns required for the hologram means that an unwanted ‘zero order’ can be present, comprising light that is not fully modulated by the SLM. This can form a high-intensity spot at the central focal position of the lens. This can be catastrophic for parallelised fabrication, as the zero-order spot intensity can be many times the intensity of the individual foci designed within the array. Therefore, techniques have been developed for zero-order removal, including a block in the Fourier plane of the SLM^127,128^, destructive interference with an additional designed spot in the array^120^ or refocusing of the focal array when carrying out surface processing^129^.

**Fig. 9:**
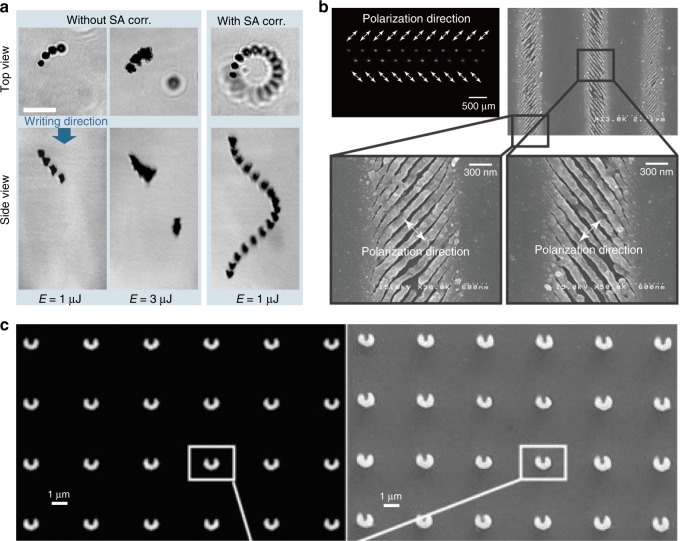
Parallel laser processing with adaptive optics and enhanced functionality. **a** Images of multispot fabrication inside the diamond bulk without aberration correction and with tailored spherical aberration (SA) compensation for each individual spot^120^. **b** Parallel fabrication with control over the polarisation of each individual focus, as evidenced by the nanograting formation^135^. **c** Parallelisation combined with beam shaping for the generation of multiple foci with complex intensity distributions^86^. Images reproduced from **a** ref. ^120^ with permission from OSA, **b** ref. ^135^ with permission from OSA and **c** ref. ^86^ with permission from AIP.

The appropriate design of a hologram enables a great level of variety and control in the form of the focal array. Foci can be arranged in three dimensions either on a regular lattice or following complex geometries^130,131^. 3D arrays of foci can be combined with aberration correction to give high-fidelity parallel fabrication deep inside challenging materials^38,120,132^, as shown in Fig. 9a. Parallelisation can be combined with beam-shaping techniques to give arrays of shaped foci^86,133,134^ and even polarisation engineering, such that each focus in the array has an independent polarisation state^135–139^, as shown in Fig. 9b. In the hologram design phase, foci can be assigned different target intensity values to give variable levels of fabrication effects across the array^125,129^. Figure 9c demonstrates parallel laser processing combined with beam shaping, such that each spot in the focal array contains an OAM to generate chiral voxels via two-photon polymerisation^86^.

Positioning holographic spots a large distance from the optic axis can be particularly problematic for ultrashort pulses, as the diffraction angle from the adaptive element is wavelength dependent, leading to a spatial spread at the focus of the different spectral components of the pulse^120^. An alternative method for generating an array of fabrication spots over a large field of view is to insert a microlens array in the fabrication beam path^10,140^, which can be beneficial, as it creates a wide array without requiring diffraction at high angles. However, the resultant spot array has a fixed spatial configuration and can experience problems related to the non-uniformity of the intensity across the array. The combination of a fixed microlens array with an adaptive element can offer a compromise by providing a route for homogenisation of the spots and tunability in the spot arrangement to generate an aperiodic array^141^.

The benefits of parallelisation mainly lie in the reduction of processing times for the fabrication of devices. To this end, focal arrays have been used in the production of large-volume gratings^128^, 3D data storage^142,143^, cell scaffolds^144^, microfluidics^133,145–147^ photonic crystals^121^, and waveguide photonic circuits^117,148–150^. Other advantages lie in the fact that distances between components can be carefully controlled, as they are fabricated simultaneously; thus, there is no potential for drift errors^117^. There can also be scientific benefits from using arrays of foci, such as for studying the light-matter interaction^125^ or the control of ion migration in glasses^151^.

**Fig. 10:**
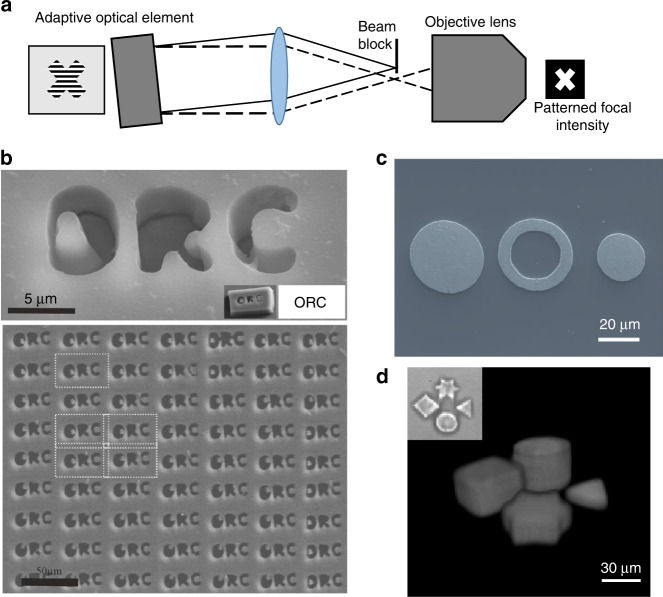
Laser processing with extended intensity distributions generated by adaptive optics. **a** Optical configuration for the projection-based creation of patterned intensity distributions using adaptive optical elements. The adaptive element is imaged onto the focal plane, and only the diffracted order is allowed to pass to the objective lens. **b** Projection-based two-photon polymerisation^157^ and **c** LIFT processing for microelectronics^154^. **d** Spatio-temporal focusing combined with adaptive optical projection for 3D photopolymerisation of complex shapes^161^. Images reproduced from **b** ref. ^157^ with permission from OSA, **c** ref. ^154^ with permission from Springer and **d** ref. ^161^ with permission from OSA.

Parallelisation using arrays of foci is not always the most appropriate way to speed up the laser writing process. In certain situations, it is better to create extended intensity distributions in desired patterns. Holography is not normally an effective approach for generating these light fields, as reconstruction noise due to the coherence of the laser leads to non-uniformity in the written structures. Although methods using a spatial or temporal dither^152^ can resolve some of the uniformity problems, they lack efficiency and can create unwanted additional material modification due to speckle. Therefore, a series of projection-based methods have been demonstrated that can provide very clean fabrication of extended structures with a single pulse. An adaptive element providing amplitude modulation can be imaged onto the workpiece, as shown in Fig. 10a, and it is thus possible to control the light distribution on the sample. As shown in Fig. 10b–d, this technique has been effective in surface processing^153^, laser-induced forward transfer (LIFT)^154–156^ and two-photon polymerisation^157,158^, using DMM devices to give potentially fast reconfiguration of the fabrication patterns. An extension combines projection with simultaneous spatio-temporal focusing (SSTF)^159,160^, which is readily achieved using DMM devices since they behave as diffraction gratings and thus automatically temporally disperse ultrashort pulses^153,161–163^.

### Temporal pulse shaping

**Fig. 11:**
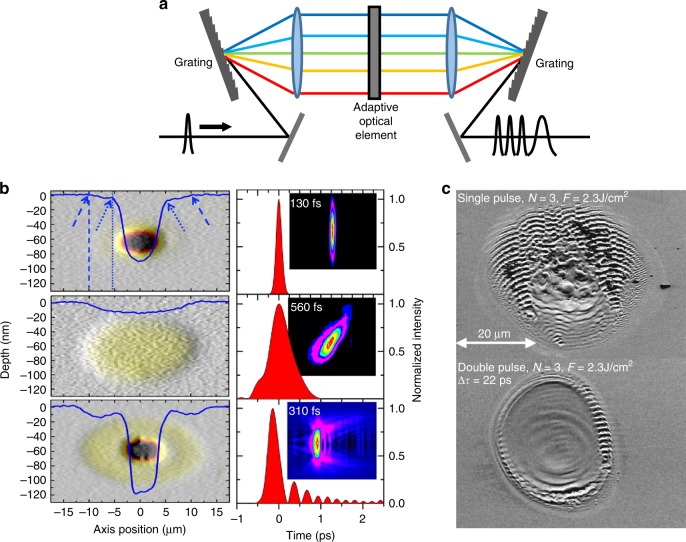
Enhanced laser processing through adaptive optics temporal pulse shaping. **a** An adaptive pulse shaper that modifies the phase of different spectral components within the pulse. Improved laser ablation of **b** sapphire^165^ and **c** silicon^166^ by respective temporal pulse shaping to tune the temporal profile of the pulse or generate a burst of pulses with short delay. Images reproduced from **b** ref. ^165^ with permission from OSA and **c** ref. ^166^ with permission from AIP.

All of the previously discussed methods concern shaping the intensity and/or phase of the fabrication laser beam in the spatial domain, giving control over the spatial intensity distribution at the focus. Important work is also being conducted on the use of adaptive optical elements for shaping the ultrashort pulsed light in the spectral domain, giving control over the temporal intensity distribution at the focus. A typical schematic is shown in Fig. 11a, where the ultrashort pulse beam is spectrally dispersed by a grating and individual spectral components focused onto an adaptive optical element, which normally takes the form of a linear SLM. The adaptive optic applies different phase values to the different spectral components before they are recombined into a beam by a second grating. Thus, it is possible to modify the temporal profile of a single pulse or turn a single pulse into a burst of separate pulses spaced closely in time^164^.

Temporally shaped pulses have been shown to be highly effective in producing improved laser ablation for a variety of materials^165–171^ by increasing material removal rates or generating smoother, more controlled modification. The ablation can be enhanced both by tailoring the temporal profile of a single pulse^165^ and by creating bursts of closely spaced pulses^166,172^, as seen in Fig. 11b, c, respectively. Of particular interest is tailoring the temporal intensity profile to complement and control the electron dynamics during the absorption process^170,173–175^. By theoretically analysing the electron dynamics during absorption and creating a bespoke temporal intensity for the fabrication beam, it is possible to substantially enhance material removal rates in laser-assisted chemical etching^176^ or increase aspect ratios in micro-channel drilling^177^.

## Discussion

There has been much progress in the use of adaptive optical elements in laser fabrication, but some challenges still exist, and more work can be done to unlock the full potential of these devices. Some of these developments will occur in concert with device development, as adaptive elements are manufactured with greater resolution, faster response and greater range. We can expect to see the extreme parallelisation of a laser beam into more distinct foci covering a greater field. As the average laser power and pulse energy increase, there is potential for laser machining with greater than 1000 simultaneous foci^126^. Optimisation is still needed, though, in hologram design for elimination of the zero order and the efficient spreading of ultrafast radiation to large angles while maintaining short pulse duration and resolution. There is further scope for the correction of larger and more challenging aberrations, pushing high-NA laser fabrication to greater depth ranges and in more complex geometries. Advances in laser beam shaping will also enable more functional structures to be fabricated in a single laser shot.

Aside from the implementations described above, adaptive elements have further functionality to offer in laser processing. Many fabrication processes have a strong dependence on the laser polarisation and can benefit from a greater level of adaptive polarisation control, including compensation for polarisation aberrations^178^. More work is also needed to merge the adaptive control of light in the spatial and temporal domains, which thus far only exist in isolation. It is known that a uniform pulse front tilt at the laser focus gives a strong directionality to the laser fabrication via the quill effect^179^, which can be manipulated through adaptive control of the laser pulse front tilt^180^. With coupled adaptive elements, it is possible to shape the laser pulsefront with the spatial resolution^181^, potentially accessing new fabrication regimes. Additionally, exploiting mechanisms such as spatio-temporal focusing^182,183^, AO can bring new dimensions to the parallelisation of individual foci in both space and time^125^. A major new avenue for AO in laser fabrication will be the combination of many different functionalities through the same instrument, such as the simultaneous combination of parallelisation, focal shaping and aberration correction.

It is also important to consider progress in laser processing as devices with refresh rates beyond the kHz level become available, with technology currently moving towards a regime where a different phase or amplitude distribution can be displayed for each fabrication pulse. This will enable fully dynamic patterning of substrates and further opportunities for optimisation of the laser process by tuning the electron dynamics in the light-matter interaction. Equally important as device development in this respect is an increased computing capability for the rapid update of displayed patterns to coincide with faster adaptive element response enabling advanced control with live feedback from the workpiece.

Further developments are also expected in the commercial sphere, where we are already starting to see the emergence of adaptive optical elements in industrial systems^184^. A possible limitation in the use of adaptive elements for laser fabrication relates to power handling capabilities. Even though the beam is not typically focused onto the device, and thus the fluence is greatly reduced relative to the fabrication focus, there is still major concern that high-average-power incident lasers can generate heating and catastrophic damage. Advances have been made to combat this issue with SLMs, where active cooling schemes are applied to the SLM back plane to minimise detrimental effects due to heating^185^. There has been significant progress in this area, with recent demonstrations reporting effective SLM operation at incident powers above 100 W^186^ as an important step for unlocking the potential of adaptive optical processes in broader laser machining applications.

## Conclusions

Adaptive optical technologies are already providing a variety of increased capabilities for the laser processing of materials. The wide range of recent developments in this area illustrates the significant potential of this technology for enabling precise fabrication inside materials through aberration correction, for manipulating the spatial distribution of light energy for structured and wide-area fabrication, and for fine control of the fabrication process. Many of these capabilities can be introduced through the incorporation of a single SLM into the optical system. As SLM technology develops further to include higher speeds and greater power handling capabilities, the range of scientific and industrial applications of adaptive optical laser fabrication is likely to expand.
